# A roadmap to reduce stunting

**DOI:** 10.1093/ajcn/nqaa205

**Published:** 2020-08-27

**Authors:** Rebecca Roediger, D Taylor Hendrixson, Mark J Manary

**Affiliations:** Department of Internal Medicine, Washington University at St. Louis, St. Louis, MO, USA; Department of Pediatrics, Washington University at St. Louis, St. Louis, MO, USA; Department of Pediatrics, Washington University at St. Louis, St. Louis, MO, USA

Stunting, defined as a low height for age z-score (HAZ), begins in the prenatal environment leading to low birth weight and continues with growth faltering in the first 2 y of life, after which it is generally irreversible. Although stunting prevalence has decreased worldwide from 1990 to 2018, stunting continues to afflict 21.3% of children aged <5 y worldwide (1). The burden of stunting falls almost entirely on low-income countries as it is tied to poverty, an excess of childhood infections, and an inadequate diet. The World Health Assembly (2) and the UN's Sustainable Development Goals (3) call for a 40% reduction in childhood stunting by 2025 with the ultimate goal of eradicating all childhood malnutrition. Although the destructive power of stunting is well known, the actions which will operationalize its reductions are not. A several part series on stunting in this issue of the *American Journal of Clinical Nutrition* (4–12) elucidates factors and policies that allow for a robust decline in stunting, by using a mixed method approach to identify how “exemplar countries” reduced stunting despite only modest improvements in economic growth.

*Stunting in childhood: an overview of global burden, trends, determinants, and drivers of decline* (4) is a systematic review of 89 studies from which basic, underlying, and immediate determinants of stunting were identified. Basic determinants are: *1*) an asset index of household income and *2*) parental education, particularly maternal education. Underlying determinants are numerous: *1*) sanitary disposal of stool, *2*) clean water, *3*) bed nets, *4*) vaccination coverage, *5*) attendance of antenatal clinic visits, *6*) optimal breastfeeding practices, and *7*) household food security. Immediate stunting determinants are: *1*) reduction in fertility, *2*) birth spacing, *3*) maternal height, *4*) infant birthweight, *5*) dietary diversity, and *6*) diarrhea incidence.

In order to understand how these determinants affected the stunting reduction in exemplar countries, an in-depth analysis of quantitative and qualitative data was employed (5). After potential exemplar countries that had a rapid decline in stunting relative to their economic growth were identified, 5 countries were studied in-depth based on a minimum population threshold of 5 million people and representation of different global regions; Peru, the Kyrgyz Republic, Nepal, Senegal, and Ethiopia.

A quantitative data analysis of each country's Demographic and Health Surveys (DHS) and Multiple Indicator Cluster Surveys (MICS), which are nationally conducted, standardized household surveys collecting health and nutrition data, was performed to discern changes in HAZ with respect to the basic, underlying, and immediate determinants. For each country, HAZ kernel density plots showing population level shifts in HAZ over time as well as changes in kurtosis, a measure of variability in the data, were prepared. Descriptive analyses evaluated inequality of HAZ changes by wealth quintile, geographic region, rural or urban setting, maternal education, and child gender. Victora curves, which plot predicted HAZ by child's age in months for each country, determined the critical ages of growth faltering as well as the maternal effect on HAZ at birth. These data formed the basis of the multivariable analyses used to identify factors that predicted an improvement in stunting.

Robust qualitative data from key stakeholders at the national, regional, and community levels were collected to gain insight into the programmatic changes and contextual factors associated with stunting reduction. These included surveys of community health workers and teachers and focus groups with mothers. This qualitative data helped to inform a timeline of key policies, programs, and practices that led to stunting reduction.

The most important data in this stunting project are the anthropometric measurements, and thus great care was taken to assess their quality (6). A composite score was created based on the proportion of collected data with incomplete demographic information or anthropometric measurements, tendency for a digit to appear more often than by chance, large differences in HAZ by month indicative of bias in age reporting, and extreme/implausible values for HAZ and weight for height z-score (WHZ). The 5 countries used for the analyses had high-quality anthropometric data, and this methodology is a tool by which other datasets can be judged.

The next 5 articles of this series are in-depth country case studies for the 5 exemplar countries, Peru, the Kyrgyz Republic, Nepal, Senegal, and Ethiopia (7–11). Baseline stunting was 25–66% and the reduction over 16–25 years was 15 to 30 percentage points (**Table 1**). Change in kurtosis in each HAZ kernel density plot was analyzed as a surrogate for changes in the distribution of stunting across the population (Table 1). A decrease in kurtosis suggests greater equity in stunting across determinant groups but could also be a marker of greater precision in data collection. Therefore, separate equity analyses were conducted to determine change in disparities among wealth quintile, education level, rural/urban setting, and gender (Table 1). Among these exemplar countries narrowing of the gap in stunting by maternal education and disparities between urban and rural settings was consistently seen.

**TABLE 1:** Summary of quantitative data for 5 exemplar countries^1^

	Senegal	Kyrgyz Republic	Ethiopia	Nepal	Peru
Study period	1992–2017	1997–2014	2000–2016	1996–2016	2000–2016
Baseline stunting prevalence	25.0%	36.2%	51%	66%	31.3%
End stunting prevalence	17.7%	12.9%	32%	36%	13.1%
Stunting reduction over study period	15%	23.3%	19%	30%	18.2%
HAZ kernel density plots					
Baseline average HAZ	−1.25 SD	−1.42 SD	−2.14 SD	−2.35 SD	−1.24 SD
End average HAZ	−0.97 SD	0.75 SD	−1.35 SD	−1.41 SD	−0.84 SD
Change in kurtosis	+0.54	+0.06	+0.04	+0.48	−0.93
Equity analysis: change in stunting between lowest/highest:					
Wealth quintile	Widened (19.6% to 20.8%)	Narrowed (27% to 10%)	Widened (12% to 24%)	Widened (22.2% to 32%)	Narrowed (46% to 27%)
Education level	Narrowed (22.6% to 8.5%)	Narrowed (13% to –1%)	Narrowed (27% to 22%)	Narrowed (39% to 25%)	Narrowed (48% to 10%)
Urban/rural	Narrowed (16% gap to 10% gap)	Narrowed (8.5% to 1.6%)	Widened (13% to 16%)	Narrowed (13% to 10%)	Narrowed (30% to 18%)
Gender gap	No change	No change	No change	No change	No change
Growth curves					
Birth length	No change (−0.4 SD to −0.4 SD)	Increased (−0.4 to 0.3 SD)	Increased (−0.4 to −0.1 SD)	Increased (−1.5SD to −0.6 SD)	No change (−0.5 to −0.6 SD)
Growth faltering 0–6 mo	Decreased	Decreased	Decreased	Decreased	Decreased
Growth faltering 6–23 mo	Decreased (−0.1 SD/mo to −0.06 SD/mo)	No change (−0.072 SD/mo to −0.073 SD/mo)	No change (−0.14 SD/mo to −0.13 SD/mo)	No change (−0.11 SD/mo to −0.081 SD/mo)	Decreased (−0.08 SD/mo to −0.03 SD/mo)

1 HAZ, height for age z-score.

Comparing the Victora curves, which plot predicted HAZ over child's age in months, across the 5 countries demonstrated 2 patterns of stunting reduction. The intercept on Victora curves is the birth length, which reflects prenatal factors, such as maternal nutrition and health. The intercept for all exemplar countries in the early 1990s was well below that of the international reference population. Ethiopia, Nepal, and the Kyrgyz Republic improved the birth HAZ over the study period, reflecting improved maternal nutrition and antenatal care. In contrast, there was no change in birth length for Peru and Senegal. The 0–6 mo time frame generally reflects breastfeeding practices and was a period of growth faltering in all countries initially. All countries demonstrated reduction in growth faltering in the 0–6 mo range suggesting improvement in breastfeeding and other practices. The 6–23 mo time frame reflects dietary practices and infectious disease management as foods and water are introduced to the infant diet during this time period. Peru and Senegal showed a dramatic reduction in growth faltering in the 6–23 mo time frame reflecting improved food security and disease prevention from improved sanitation practices. In contrast, Ethiopia, Nepal, and the Kyrgyz Republic showed much less improvement in growth faltering in the 6–23 mo time period. These data suggest that there are multiple time periods that can serve as effective targets when attempting to reduce stunting prevalence. All 5 exemplar countries reduced their stunting prevalence but from this data it appears that Senegal and Peru accomplished this by preventing growth faltering from 6–23 mo with no improvements in birth length, in contrast to the other exemplar countries which accomplished stunting prevalence reduction via marked improvements in birth length but no change in growth faltering at 6–23 mo (Table 1).

Determinants for the reduction in stunting were identified for each country by multivariable analysis of the health survey data and by qualitative data collection from key stakeholders. The multivariable models explained 72–100% of the improvement in mean HAZ depending on the exemplar country. Senegal, Nepal, and the Kyrgyz Republic did not have national data on food security, which likely contributed to the larger fraction left unexplained by the multivariable model.

Although there were a few determinants of stunting reduction that were specific to certain countries (e.g., migration from the mountainous regions in Peru; higher crop yield in Ethiopia), there were many determinants that were important across all exemplar countries (**Table 2**). The authors classified the determinants as nonhealth sector improvements and health sector improvements (12). They found that nonhealth sector improvements, such as government programs for poverty relief, maternal education, and agriculture changes accounted for 36–70% (median 47%) of stunting reduction. Health sector changes such as maternal and newborn health care, access to family planning/reduction in fertility, and maternal nutritional status accounted for 20–64% (median 37%) of changes in HAZ. The qualitative analysis identified key programs and policies that led to improvements in these sectors, and the focus groups with mothers in the community confirmed which programs affected change at the household level.

**TABLE 2:** Changes in potential determinants for reducing stunting prevalence over the study period

	Senegal	Kyrgyz Republic	Ethiopia	Nepal	Peru
Multivariable analysis for children under 5 y					
Variability explained by multivariable model	72%	88.9%	110%	90.9%	109%
Nonhealth sector					
Wealth index, 0–10	+0.91^1^	+0.63^1^	+0.85^1^	+1.11^1^	−0.23^1^
Open defecation, % of population	−23.9%	—	−50.3%^1^	−54.7%^1^	−17.4%
Clean water, % of population	+25.2%^1^	+9.61%	+14.9%	+13.6%	+7.7%
Maternal education, y	+1.69^1^	+30%	+1.22^1^	+3.63^1^	+2.13^1^
Duration of breastfeeding, mo	−2.2	−1.1	−3.17^1^	−1.45^1^	+0.55^1^
Health sector changes					
Respiratory illness prevalence, % under 5 y	−11.6%	−10.4%	−15.2%	−23.4%	−9.2%
Diarrhea prevalence, % under 5 y	−3.7%	−11.9%	−14.4%	−15.3%	−4.6%^1^
Maternal BMI, kg/m^2^	—	+1.08^1^	+0.62^1^	+1.5^1^	+1.51^1^
Antenatal visits ≥4, % pregnant women	+43%^1^	—	+21.3%^1^	+54.8%^1^	+26.8%^1^
Birthweight <2500 g, %	+3.2%	+0.58%^1^	+5.36%	—	−1.9%^1^
Maternal age, y	+1^1^	+0.77^1^	−0.15^1^	−1.2^1^	+0.8^1^
Fertility, children per mother	−0.77^1^	−0.35^1^	−0.24^1^	−1.05^1^	−0.65^1^

^1^Significant determinants of height for age z-score change over time identified in multivariable modeling.

From this 9-part stunting series, key drivers of stunting reduction were elucidated using a robust mixed methods approach which can be applied to other low- and middle-income countries (LMICs) aiming to reduce stunting prevalence. In all countries, factors that were identified in the multivariable analysis as significant contributors to stunting reduction were improvements in poverty, maternal education, maternal nutrition status, good antenatal care, increase in maternal age, and reduction in fertility (Table 2). Although a reduction in poverty, measured by change in wealth index, was significant in all countries, exemplar countries were picked because their stunting reduction was out of proportion to their economic gains. Therefore, the other factors that were found to be significant across all countries should act as targets for other countries aiming to reduce the burden of stunting. This body of work is important as it can serve as a template for ways to reduce stunting burden in other countries. Both by elucidating common factors between all 5 exemplar countries and by highlighting the mixed methods approach, this body of work will allow other countries to study the efficacy of their national programs on stunting reduction.

This in-depth analysis of 5 exemplar countries presents a road map for how to reduce stunting prevalence. However, many LMICs struggle with a high burden of stunting and have employed programs of their own to attempt to decrease the burden. This series does not compare effective programs from the exemplar countries with similar but failed programs in other countries. It would be helpful to contrast exemplar countries with countries who have not been able to reduce their stunting prevalence in order to identify what specific elements of the government and nongovernmental organizations programs in exemplar countries allowed for success when compared to similar programs that are applied worldwide with less impact.

Even within these 5 exemplar countries more contrasts could have been identified to guide future programs. For example, 2 patterns emerged from the Victora curves, 1 pattern showing increases in birth length but similar growth faltering at 6–23 mo and the other showing no changes in birth length but greater reduction of growth faltering in the 6–23 mo range. This series does not contrast the programs that were rolled out in each country to deliver these different patterns of stunting reduction which would be helpful guidance for other countries looking to emulate their success.

Many countries implemented micronutrient supplementation programs over the course of the study period, which are identified in literature reviews and qualitative analysis of national and regional stakeholders as being important drivers for stunting reduction. However, the actual effects of micronutrient supplementation on changes in HAZ were not conducted in their quantitative analysis as the data on these programs was not routinely collected in the national surveys. Finally, a cost-effectiveness analysis would provide more information about which programs had the greatest impact for dollar spent and would allow countries looking to emulate the exemplar country's stunting reductions to know where best to focus their efforts and budget.

This body of work and future directions are especially important as the COVID-19 pandemic is predicted to worsen malnutrition globally. It is estimated that the prevalence of wasting could increase 10–50% causing an excess of ≤2 million child deaths (13). The required self-isolation and country-wide lockdowns alongside the commensurate change in focus of the health care system and worsening economic conditions will have myriad downstream effects on health and nutrition, especially in LMICs (14). The disruption to food supply chains coupled with decreases in household income will compound food insecurity. Households will rely on less nutritious but easily accessible and cheaper processed foods. The limited access to health care due to travel restrictions and health center's shift in focus to COVID-19 will reduce access to family planning and prenatal care. In addition, the reduced financial resources will disrupt the social safety net and programs intended to identify and treat malnutrition. Globally, schooling has been interrupted due to coronavirus concerns which prohibits school-based nutrition initiatives in the short term and will also have long-term effects as maternal education is tied with stunting reduction. Finally, water/sanitation/hygiene projects will be put on hold during the pandemic but remain especially important in urban crowded settings under lockdown as outbreaks of communicable diseases are common without adequate water and sanitation. As the current pandemic is predicted to worsen malnutrition worldwide, this body of work remains of paramount importance to guide countries as they work to implement programs to mitigate the effects of the pandemic on their population.

In sum, it is possible for substantial reductions in stunting to be realized. In the exemplar countries there was careful accounting for the local context, which allowed for mobilization of existing resources. As with all great endeavors, champions are required to overcome the natural entropic tendencies. Given the future dividends that will accrue with reduced stunting, embrace of this sustainable development goal is worthy wherever stunting exerts it scourge.

## Notes

The authors reported no funding received for this work.
